# Atypical presentation of angiosarcoma of the scalp in the setting of Human Immunodeficiency Virus (HIV)

**DOI:** 10.1186/1477-7819-7-99

**Published:** 2009-12-18

**Authors:** Poovandren S Govender

**Affiliations:** 1 Department of Radiotherapy and Oncology, Nelson R. Mandela School of Medicine, University of Kwazulu Natal, Durban, South Africa

## Abstract

**Background:**

Angiosarcoma of the head and neck is an uncommon, aggressive malignant entity most commonly found in elderly Caucasian males. We present a case in a young black female with co-existing HIV. The atypical gender, age and race of the patient reflect the unusual clinical presentation of this case of angiosarcoma, attributable to the patient's HIV status.

**Case presentation:**

A 22 year old patient presented with a large unresectable lesion over the occiput with surrounding ulceration, satellite lesions and associated lymphadenopathy. She is HIV-infected with a CD4 count of 360 cells/μl. She was not on antiretroviral treatment based on South African treatment guidelines advocating antiretroviral treatment when the CD4 count is below 200 cells/μl, in the absence of other AIDS-defining illnesses.

The patient was treated with a course of ifosfamide and anthracyline based chemotherapy. Disease progression was noted on chemotherapy and she was subsequently palliated with a course of radiotherapy. She had a satisfactory response with an improvement in local symptoms. She is currently receiving symptomatic care.

**Conclusions:**

South Africa is at the epicenter of the HIV epidemic. Consequently, the management of patients in the field of oncology in our clinical practice is often burdened with malignancies manifesting with an atypical disease presentation and clinical course.

## Background

Angiosarcoma of the head and neck is an uncommon aggressive cancer of the skin and soft tissues [1]. These malignancies are most commonly found in Caucasians and are 3-4 times more common in males than females with a median age of 61 to 67 years [2,3].

We report an atypical presentation of a case of angiosarcoma of the scalp occurring in association with HIV in a 22 year old black female.

Linkage studies of population-based registries involving people with HIV/AIDS and cancer have shown a statistically significant increase in the incidence and relative risk of many Non-AIDS Defining Cancers (NADCs). Tumours displaying unusual features and aggressive behaviour patterns in young individuals should alert physicians to the possibility of underlying HIV infection [4].

HIV-related defects in the cell mediated immunity lead to an increased risk of malignancy due to decreased tumour surveillance and suppression of oncogenic viruses. However, the role of immunosuppression in the pathogenesis of NADC is controversial, based on studies demonstrating that the increased risk of NADC is not associated with low CD4 T-lymphocyte cell counts or the onset of AIDS [4].

The impact of HIV on the South African healthcare system has been profound. The total number of persons living with HIV in South Africa increased from an estimated 4.1 million in 2001 to 5.2 million by 2009. For 2009, an estimated 10.6% of the total population is HIV positive [5].

The scourge of HIV in sub-Saharan Africa has contributed to the unusual clinical presentation and natural history of malignant disease.

## Case presentation

A young black female presented with a 6 month history of a mass over the posterior aspect of her scalp which was initially thought to be an abscess and subsequently ulcerated. There was no history of previous radiotherapy or trauma to the scalp.

**Figure 1:**
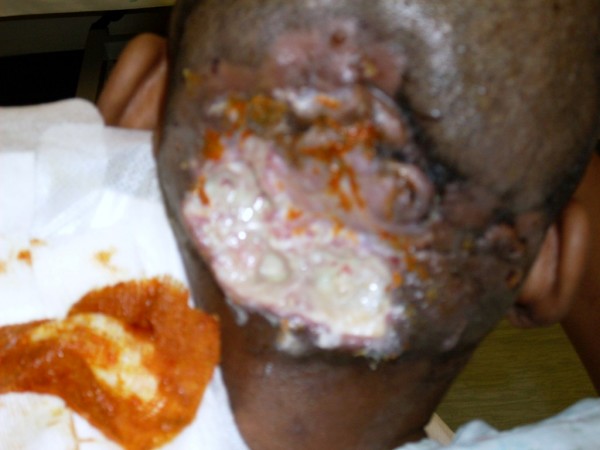
**Initial presentation**.

Clinically, she had a 12 cm by 10 cm diameter, ulcerative lesion over the occiput with surrounding ulceration and satellite lesions. Examination of the neck revealed bilateral level II-V lymphadenopathy measuring approximately 1 cm in diameter (Figure 1).

Biopsy of the occipital scalp mass demonstrated an epithelioid angiosarcoma. She also had a biopsy of a cervical lymph node which demonstrated features of a metastatic epithelioid angiosarcoma.

HIV Elisa was positive with a CD4 count of 360 cells/μl.

**Figure 2:**
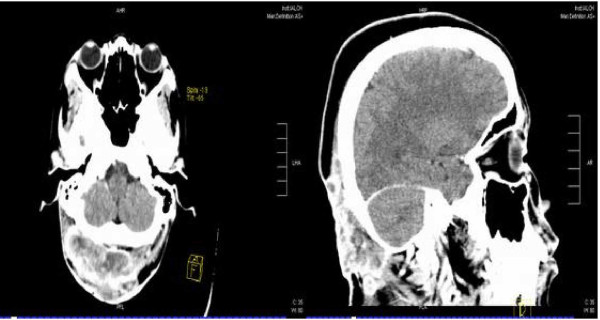
**Enhancing mass lesion in occiput on axial and saggital CT slices**.

Computed Tomography (CT) scan revealed a large heterogeneously enhancing soft tissue mass over the scalp in the occipital region with erosion of the underlying bone. Extensive subcentimetre enhancing lymphadenopathy was noted in the anterior and posterior triangles of the neck with the largest noted in the right parotid region measuring 1.1 cm. No distant metastases were noted (Figure 2).

**Figure 3:**
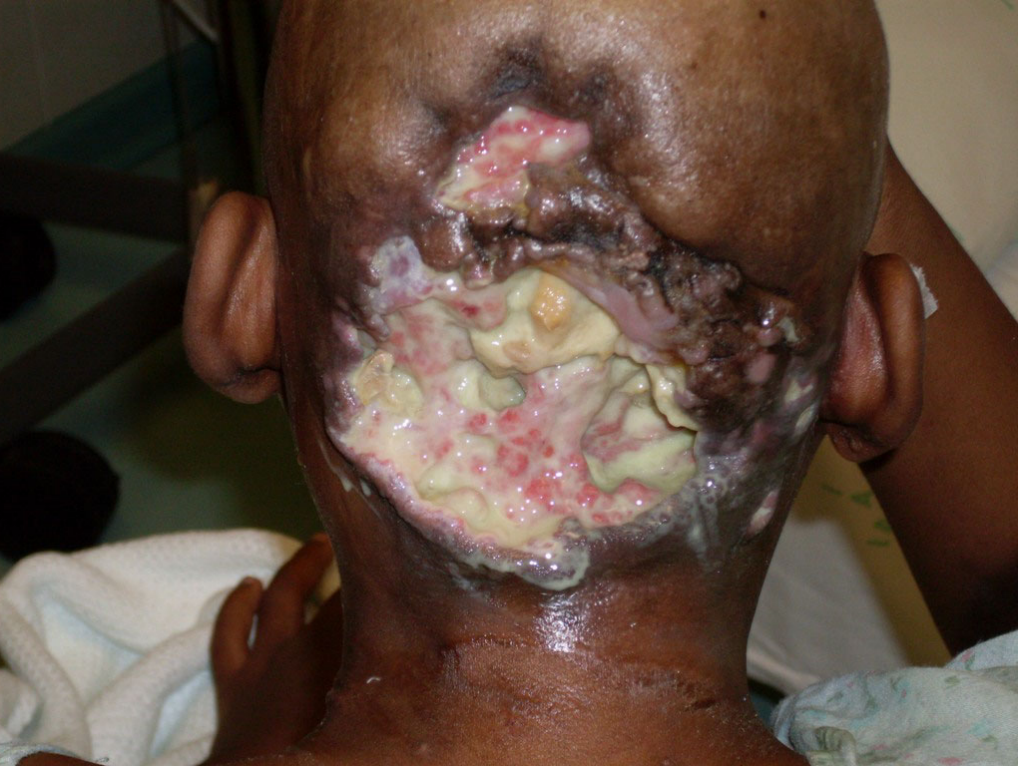
**Disease progression on chemotherapy**.

She was treated with chemotherapy (Epi-doxorubicin 40 mg/m^2 ^I.V d1-3, Ifosfamide 1.5 g/m^2 ^I.V d1-3, q 3/52). Clinical disease progression was noted after 3 cycles of chemotherapy (Figure 3).

We then opted to treat her palliatively with electron beam radiotherapy. An irregular electron cut-out was used to define the treatment area. A single posterior field of 15 MeV electrons was used to deliver the prescribed dose of 3000 cGy to the 80% isodose curve in 10 fractions (3 Gy/fraction) over 2 weeks. She had a satisfactory response to treatment with an improvement in local symptoms.

She was subsequently managed symptomatically.

## Discussion

Sarcomas are uncommon in the head and neck region constituting only 1% of all malignant neoplasms in this region. Angiosarcomas make up less than 1 percent of all sarcomas. Their most frequent site of origin is the head and neck, especially the scalp of elderly men. These highly aggressive mesenchymal tumours may arise in association with some recognised clinical condition (chronic lymphoedema or previous irradiation) or as a de novo occurrence [6].

Cutaneous angiosarcoma of the head and neck is a distinct subtype which most commonly presents as an enlarging purple bruise-like lesion that develops over several months. There may be intermittent bleeding, oedema or ulceration. A delay in diagnosis is common in the early stages of disease due to confusion with infection, or traumatic bruises [7].

These malignancies spread radially within the dermis of the scalp and face and are associated with ecchymotic patches extending beyond the obvious lesion, making it difficult to achieve excision with clear margins. It is often multifocal and associated with a high incidence (10-15%) of lymph node metastasis [2].

There is currently no accepted staging system for this disease [8].

There is also no standard treatment schedule for these malignancies on account of its rarity. Surgery is considered the mainstay of treatment with wide local excision and frozen section control. Prognosis correlates well with the ability to attain clear surgical resection margins although the tendency of scalp angiosarcoma to demonstrate a diffuse pattern of clinically undetectable spread makes resection challenging [9].

The role of radiotherapy is less well-defined. The reported outcomes of radiotherapy alone have largely been unsatisfactory. Several authors have thus recommended a treatment approach comprising both surgery and radiotherapy. Wide field radiotherapy appears to be a rational therapeutic choice for scalp angiosarcoma because the involved dermis as well as a sufficient area of surrounding skin can be treated, while sparing the brain and other normal tissue [9].

Chemotherapy has been suggested for unresectable cases, but has generally not proven beneficial [8]. Anthracyclines have been most commonly used in the past, either as a single agent or as a component of combination therapy [7]. Although no phase II trials were performed specifically for angiosarcomas, doxorubicin and ifosfamide are generally considered the most active chemotherapeutic agents [10]. Liposomal doxorubicin and paclitaxel have also demonstrated response in angiosarcomas [1-10].

The overall prognosis for angiosarcomas is poor when compared to other head and neck sarcomas with the 5 year survival rate ranging from 4 to 20% [6]. Local recurrence and metastases are very frequent regardless of the treatment modality employed [8].

## Conclusion

South Africa is at the epicenter of the HIV epidemic. Consequently, the management of patients with malignant neoplasms in our context is often compounded by atypical disease presentation and clinical course. This case of angiosarcoma of the scalp co-existing with HIV is indicative of the challenges faced by the oncology fraternity in our country.

## Consent

Written informed consent was obtained from the patient for publication of this case report and any accompanying images. A copy of the written consent is available for review by the Editor-in-Chief of this journal.

## Competing interests

The author declares that they have no competing interests.

## Authors' contributions

PSG participated in the treatment of the patient, collection of case details, literature search and drafted the manuscript. The author has read and approved the final manuscript.
